# A truncating variant altering the extreme C-terminal region of desmoplakin (*DSP*) suggests the crucial functional role of the region: a case report study

**DOI:** 10.1186/s12920-023-01527-6

**Published:** 2023-05-04

**Authors:** Malena P. Pantou, Polyxeni Gourzi, Vasiliki Vlagkouli, Efstathios Papatheodorou, Alexandros Tsoutsinos, Eva Nyktari, Dimitrios Degiannis, Aris Anastasakis

**Affiliations:** 1 Molecular Immunopathology, Histocompatibility and Genetics Laboratory, Onassis Cardiac Surgery Center, Kallithea, Greece; 2 Unit of Inherited and Rare Cardiovascular Diseases, Onassis Cardiac Surgery Center, Kallithea, Greece; 3 Department of Pediatric Cardiology, Onassis Cardiac Surgery Center, Kallithea, Greece; 4 CMR Unit, Onassis Cardiac Surgery Center, Kallithea, Greece

**Keywords:** *DSP*, Arrhythmogenic cardiomyopathy, Mutation, Case report, C-terminal region

## Abstract

**Background:**

Homozygous truncating mutations located in the C-terminal region of the desmoplakin gene (*DSP*) are known to mainly cause Carvajal syndrome, an autosomal recessive syndromic form of arrhythmogenic cardiomyopathy with an extra-cardiac cutaneous phenotype.

**Case presentation:**

Here we describe a female proband with a documented arrhythmogenic left ventricular cardiomyopathy and a syncopal episode at the age of 13, who was found homozygous for the novel *DSP* variant: NM_004415.4:c.8586delC, p.(Ser2863Hisfs*20) at the extreme C-terminal region of the protein, just 8 amino acids upstream the stop codon. She did not have any of the typical dermatological symptoms that characterize Carvajal syndrome. Her brother had died suddenly at the age of 18 during exercise and was found homozygous for the same variant at the post-mortem, while their parents were heterozygous. The region of origin of both parents was the same geographic area of Greece, but they were not aware of any common ancestor. Detailed clinical examination revealed that the mother displayed a mild arrhythmic phenotype, while the father was asymptomatic.

**Conclusion:**

These observations pinpoint to a significant functional role of the extreme C-terminal tail of the protein.

## Background

Arrhythmogenic cardiomyopathy (ACM) is a disorder characterized by an early propensity to symptomatic arrhythmia, initially disproportionate to the degree of ventricular dysfunction, with subsequent deterioration of ventricular function [1]. ACM was once viewed as a disease of the right ventricle (RV) with only minor or late-onset involvement of the left ventricle (LV). However, in recent years, the ACM clinical spectrum has grown to include both LV- (i.e., arrhythmogenic left ventricular cardiomyopathy [ALVC]) and biventricular-predominant patterns of disease [2, 3]. Additionally, a growing body of evidence has demonstrated that ACM can present during both childhood and adolescence, although it was once regarded as a disease of young adults (i.e., individuals in their third or fourth decade of life) [4]. Arrhythmogenic cardiomyopathy is mainly associated with mutations in desmosomal genes [5–9].

Desmosomes are major cell adhesion junctions prominent in the epidermis and myocardium, serving as links between intermediate filaments (IFs) and the cell membrane in adjacent cells and contributing to the tissue architecture and integrity [10]. The desmosomes consist of several proteins of whom the most abundant is desmoplakin, a protein encoded by the *DSP* gene. Its main function is the anchoring of intermediate filaments to desmosomes.

Mutations in *DSP* (MIM#125647) may manifest as Carvajal syndrome, lethal acantholytic epidermolysis bullosa, skin fragility–wooly hair syndrome, striate palmoplantar keratoderma, and other phenotypes involving hair, nails, and skin, while early cutaneous findings may herald future cardiac involvement [11]. The Carvajal syndrome is an autosomal recessive syndromic form of ACM (MIM#605676) with an extra-cardiac cutaneous phenotype mainly caused by homozygous truncating mutations located in the C-terminal region of *DSP* [6, 12]. Initially, the cardiac phenotype in Carvajal syndrome resembled to dilated cardiomyopathy [13], but recent clinical data from 107 patients indicate that *DSP* cardiomyopathy is a distinct form of arrhythmogenic cardiomyopathy characterized by episodic myocardial injury, left ventricular fibrosis that precedes systolic dysfunction and a high incidence of ventricular arrhythmias [14].

In this study, we report for the first time a female patient carrying an homozygous frameshift mutation at the extreme C-terminal region of the protein, just 8 amino acids upstream the stop codon, manifesting a highly arrhythmic profile and the characteristic LV involvement but without any dermatological symptoms.

## Case presentation

### Patients

**Fig. 1:**
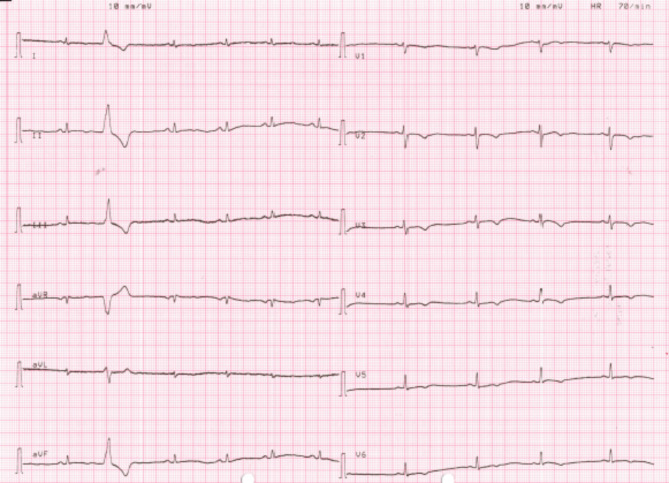
Proband’s (II2) resting ECG: T-wave inversion in leads V1-V6, flattened T waves and low QRS voltages (< 0.5 mV) in limb leads and a single VPB are noted

**Fig. 2:**
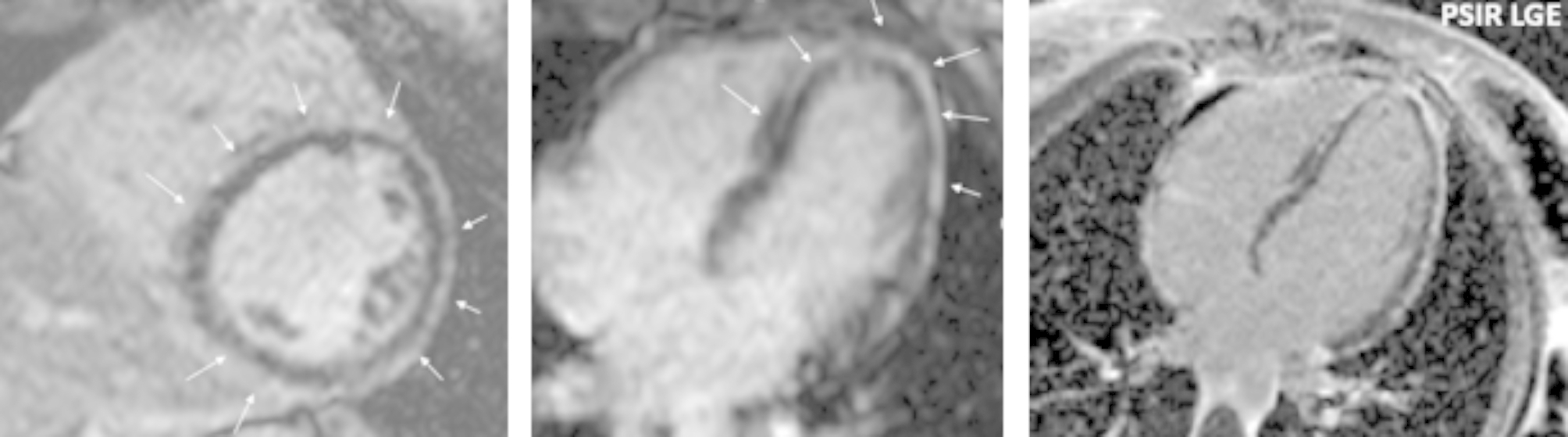
Proband’s CMR (II2): Extensive subepicardial late gadolinium enhancement (LGE) with a circumferential ring-like pattern in the LV (white arrows)

**Fig. 3:**
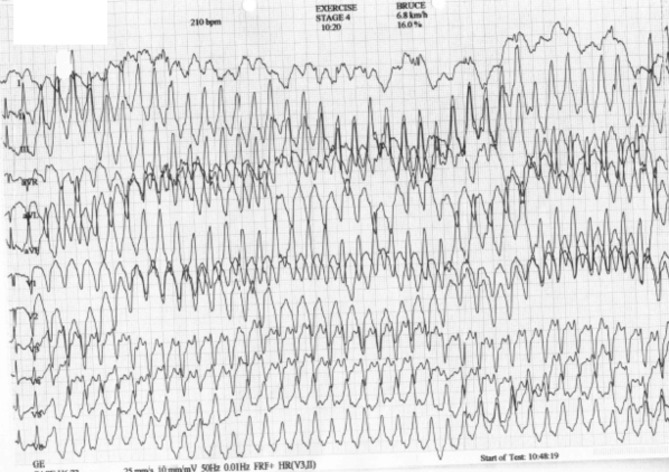
Proband’s exercise test (II2): ventricular tachycardia with a LBBB with inferior axis morphology, suggesting a RV outflow tract origin

The proband was a 13-years old female athlete who was evaluated due to a syncopal event. Her ECG showed T-wave inversion in V1-V4 leads and low QRS voltages in the limb leads. The echocardiogram was within normal limits and no further evaluation was requested at the time. She was re-evaluated at the age of 15, after the sudden cardiac death (SCD) of her brother. She reported no further symptoms. Her resting ECG showed extensive repolarization abnormalities with T-wave inversion in leads V1-V6, flattened T waves and low QRS voltages in the limb leads, as well as ventricular premature beats (VPBs) (Fig. 1). Her echocardiogram revealed mild biventricular dilatation with borderline biventricular function (LV ejection fraction of 50%). There were no RV dyskinetic areas or aneurysms. Cardiac magnetic resonance (CMR) showed extensive circumferential subepicardial LV late gadolinium enhancement (LGE) compatible with a ring-like myocardial fibrosis that affected 35% of the LV myocardial mass (Fig. 2). There was LGE in the inferior RV wall. There was no evidence of inflammation. She displayed a highly arrhythmic profile with 7000 polymorphic VPBs with both a left bundle branch block (LBBB) and right bundle branch block (RBBB) morphology in a 12-lead 24-hours Holter monitoring. In the exercise test, she developed a symptomatic sustained ventricular tachycardia with an LBBB morphology, inferior axis and late precordial transition suggesting an RV outflow tract origin (Fig. 3). Based on these findings she was diagnosed with ALVC according to the Padua Criteria [15, 16] (Table 1). She was classified at high risk of SCD and an ICD was implanted. One week after the implantation, the ICD was appropriate discharged.

**Table 1:** Clinical, electrocardiographic, echocardiographic and cardiac magnetic resonance data of the proband and her family members

Patient	Age (years)	ECG	Echo study	CMR	24-hours Holter/ Exersice test	Outcome	*DSP* mutation status
**Proband II2**	13	T-wave inversion in anterior leads. Low QRS voltages in limb leads	normal	Not tested	Not tested/ Not tested	Syncope	hom
	15	T-wave inversion anterior and lateral leads. Low QRS voltages in limb leads.	Mild biventricular dilatation. LVEF: 50%	Extensive ring-like subepicardial LGE of the LV and RV LGE: 35% of the LV mass	7000 polymorphic VPBs/ Sustained ventricular tachycardia	Diagnosis of left-dominant ACM, ICD implantation, ICD appropriate discharge	
**II1**	18	Non-specific T wave abnormalities, polymorphic VPBs	normal	Not tested	Not tested/ Not tested	Died during exercise. Post-mortem: LV fibrosis	hom
**I2**	51	Non-specific T wave abnormalities, VPBs	normal	normal	20.000 VPBs/ Not tested	mild sense of palpitations	het
**I1**	52	normal	normal	Not tested	Not tested/ Not tested	Asymptomatic	het

**Fig. 4:**
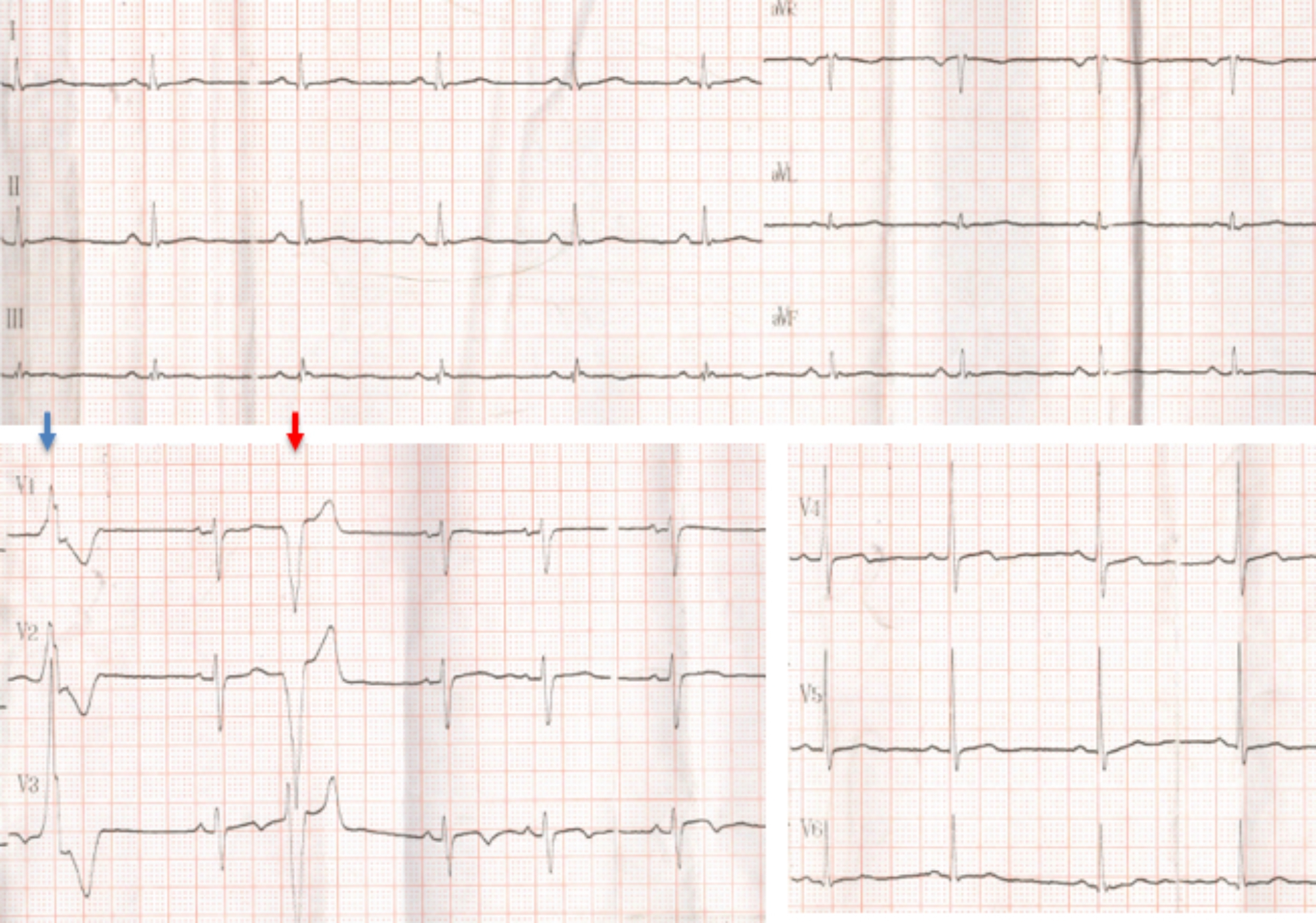
ECG of the proband’s brother who died suddenly during exercise (II1): Non-specific T wave abnormalities with T-wave inversion in lead V3 and polymorphic VPBs. Two different morphologies are noted: RBBB - indicating origin from the left ventricle (blue arrow) and LBBB - indicating origin from the right ventricle (red arrow)

Her brother was an athlete without any symptoms who died suddenly during exercise at the age of 18-years. In the athletic pre-participation cardiovascular screening he had a pathological resting ECG with an isolated negative T wave in lead V3 and polymorphic VPBs originating from both ventricles (Fig. 4). His echocardiogram was within normal range and no further cardiac evaluation was requested. The post-mortem examination revealed areas of LV fibrosis.

The mother was 51 years old and complained of palpitations. Her ECG showed an isolated T negative T wave in lead aVL and RVOT originated VPBs. Upon cardiovascular imaging there was mild RV dilatation without any regional wall motion abnormalities or evidence of myocardial fibrosis. She exhibited more than 20,000 VPBs per 24 h without evidence of complex ventricular arrhythmias. The father, 52 years old, was asymptomatic with a normal ECG and echocardiogram and with no evidence of arrhythmias.

### Genetic analysis

**Fig. 5:**
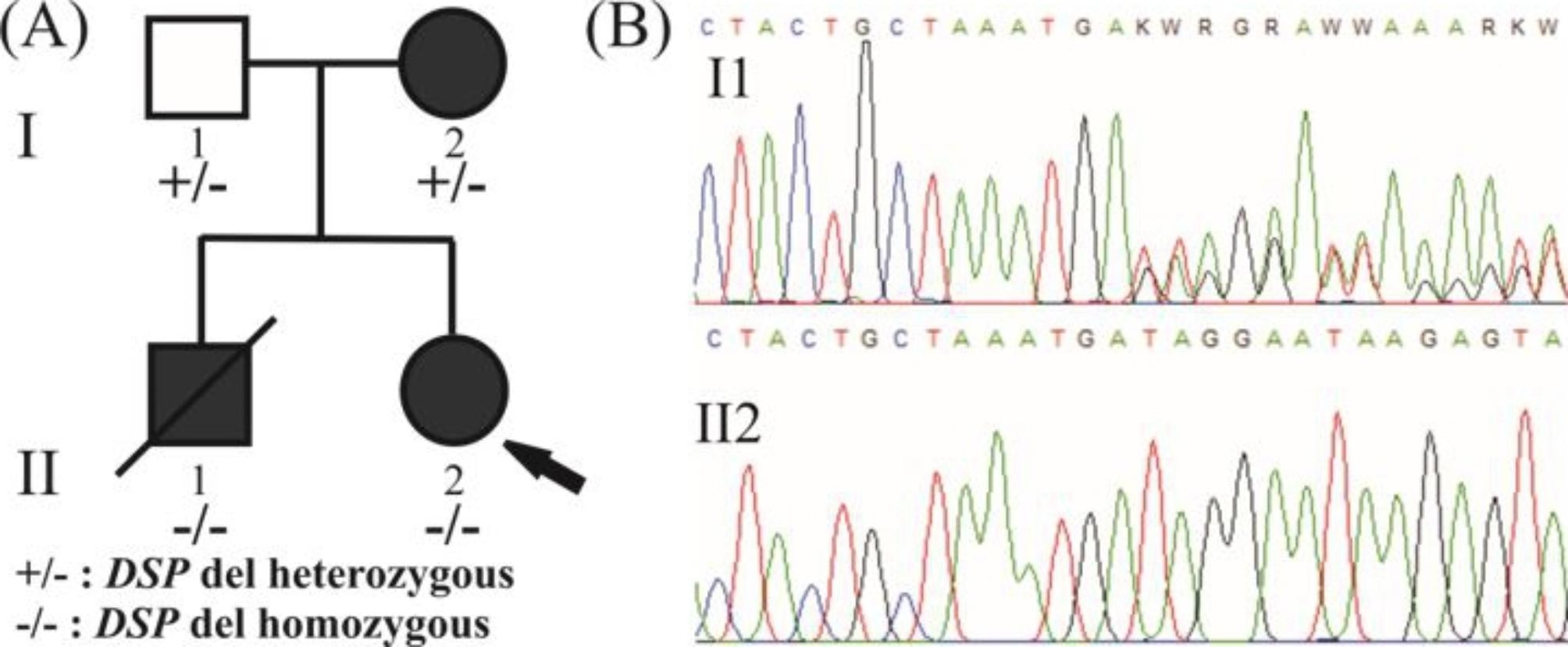
(A) The family pedigree is depicted. The proband (shown by arrow, II2) was homozygous for the NM_004415.4:c.8586delC. Her brother (II1) died suddenly during exercise and was also homozygous for the same variant. The proband’s parents (I1 and I2) were found heterozygous for the same variant. Circle = female. Square = male. Filled symbols = affected individuals. (B) Electropherogram of the involved sequence fragment of the *DSP* for the members of the family

The molecular basis of the disease was identified for the proband with next generation sequencing technology, using Illumina’s Trusight Cardio sequencing panel, covering 174 genes clinically relevant to cardiac diseases. Alignment, quality filtering, variant calling and variant annotation were performed using in parallel the standard MiSeq Reporter (Illumina) and the Sophia Genetics pipeline. The variant calling files were filtered using the Sophia Genetics DDM platform and the detected variants were characterized according to the recommendations of the American College of Medical Genetics and Genomics (ACMG) [17]. All benign or likely benign variants were filtered out and the retained variants (Table 2) were then evaluated according to the relevance of the gene to the observed phenotype resulting in one plausible candidate variant which was located in the *DSP* gene: NM_004415.4:c.8586delC,p.(Ser2863Hisfs*20). The proband was homozygous for the variant (https://databases.lovd.nl/shared/individuals/00424916), which was novel and characterized as VUS (variant of unknown significance) (according to ACMG criteria). It was a frameshift variant located in the last exon of the gene producing a transcript that was not predicted to undergo nonsense-mediated mRNA decay. Specifically, the variant resulted in the substitution of the last 9 amino acids of the protein and the prolongation of the C-terminal tail by 10 additional amino acids (PVS1 criterion reduced to moderate; [18]). Furthermore, it was absent from the population databases of Exome Sequencing Project and Genome Aggregation Database (PM2 criterion). The parents of the proband underwent *DSP* genetic testing by targeted Sanger sequencing for the detection of the variant and were found both heterozygous for the same variant. Material from the autopsy subjected to genetic testing revealed that the proband’s brother was also homozygous for the same variant. Following these genetic results and during the course of a second and more detailed interview, the parents revealed a common origin from the same geographic region of Greece, but they were not aware of any common ancestor (Fig. 5).

**Table 2:** List of VUS variants observed in the proband

GENE	OMIM	Mutation	Gnomad	ClinVar
*DSP*	Arrhythmogenic right ventricular dysplasia 8 (MIM#607450), Cardiomyopathy, dilated, with woolly hair and keratoderma (MIM#605676)	NM_004415.4:c.8586delC p.(Ser2264Hisfs*20)	Not found	Not reported
*DOLK*	Congenital disorder of glycosylation, type Im (MIM#610768)	NM_014908.4:c.247C>T p.(Pro83Ser)	Not found	Not reported
*LAMA2*	Muscular dystrophy, congenital, merosin deficient or partially deficient (MIM#607855), Muscular dystrophy, limb-girdle, autosomal recessive 23 (MIM#618138)	NM_000426.4:c.9262A>G p.(Ile3088Val)	3.5e-05	VCV000968551.4 VUS (2 submissions) LB (1 submission)
*PRKAR1A*	Carney complex, type 1 (MIM#160,980), Acrodysostosis 1, with or without hormone resistance (MIM#101800)	NM_212471.3:c.973+48C>T	7.0e-06	Not reported

## Discussion and conclusions

Desmosomes are intercellular junctions found in epithelial and cardiac tissue. They mediate the connection of IFs of neighboring cells, creating a network of adhesive structural interactions conferring strength and durability to these tissues. Cardiac IFs consist mainly of desmin and mutation in *DES* gene can cause ACM [19]. Desmoplakin is an essential component of desmosomes as it serves the connection of the inner desmosomal plaque comprised of the cytoskeletal IFs to the plakoglobin and plakophilin molecules of the outer desmosomal plaque [20]. A modular structure with distinct domains of the protein interacting with neighboring molecules is the hallmark of desmosomal proteins. In the case of desmoplakin, the plakin domain in the N-terminal region of the protein interact with plakophilin and plakoglobin [21], the central coiled-coil region is responsible for desmoplakin dimerization [22], while three plakin repeat domains (PRDs A, B, and C) in the C-terminal region of the protein are reported to bind to IFs [21, 23–25].

**Fig. 6:**
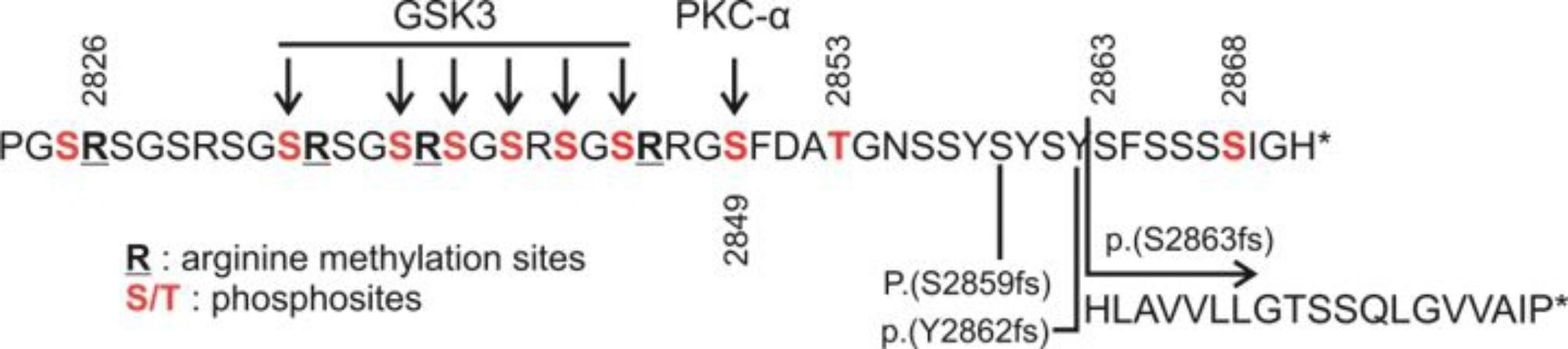
Schematic diagram of arginine methylation sites (bold and underlined) and serine/threonine sites (red) phosphorylated by PKC-ɑ and GSK3 within the C-terminal extremity of desmoplakin (aa 2823–2871). The change introduced by the mutation of the proband p.(S2863fs) is depicted along with two other frameshift mutation that have been observed in this region

Beyond PRD-C, in the carboxyl extremity of the protein, several amino acid residues, comprising the Gly-Ser-Arg (GSR) repeats, undergo post-translational modifications (Fig. 6). Such modifications of cell junction proteins by phosphorylation and methylation represent a specific, rapid mechanism for regulating their function and association with neighboring molecules [26–28]. Furthermore, the carboxyl extremity has been proposed to form an arginine claw [28], a recently identified structural element of serine-rich regions. This was first characterized in the C-terminal region of ASF/SF2 [29], a protein involved in mRNA splicing, spliceosome assembly, and mRNA nuclear trafficking. This structure is affected by the post-translational modifications observed in the region and this structural interplay may function as a molecular switch for the binding affinity of desmoplakin to the IFs [28].

In this context, an amino acid residue that has been shown to undergo post-translational modification is serine 2849 (Fig. 6), which is phosphorylated by PKC-ɑ [30]. This phosphorylation reportedly induce conformational changes that permit the recruitment of GSK3, a second kinase, that further phosphorylates upstream located serine residues (positions 2833–2845, [27]). Other nearby residues that have been found phosphorylated are Thr2853 and Ser2868 [31]). So far, two frameshift mutations have been reported in ClinVar downstream of Ser2849:p.(Ser2859fs) (rs727504909) and p.(Tyr2862fs) (rs765683790) in patients with clinical phenotype of ACM and have been characterized as VUS, since they are not anticipated to result in nonsense mediated decay and due to the lack of evidence on the functional role of the extreme C-terminal 20 residues (database assessed April 11, 2023). Camors et al. [32] described an homozygous case for the p.(Ser2859fs) variant presenting infantile epidermolysis bullosa and severe ACM, who died at the age of 12 months. It was the child of a consanguineous marriage, whose mother reportedly died prematurely from hypertrophic cardiomyopathy and whose father had lifelong lesions on his feet but refuted genetic testing. Our patient carried a homozygous frameshift mutation at position 2863 altering the last 9 amino acids of the protein and prolongating the C-terminal tail by 10 additional amino acids (Fig. 6). She displayed a malignant cardiac phenotype with significant biventricular fibrosis, extensive T wave inversion, diffuse and a highly arrhythmic profile but without any dermatological symptoms. Her homozygous brother had VBPs in his resting ECG, died suddenly during exercise and the post-mortem revealed LV fibrosis. The heterozygous mother (aged 51) displayed milder arrhythmia symptoms, while the heterozygous father was asymptomatic (Table 1). This phenotype is suggestive of a functional role of this extreme C-terminal region, as the mutation might perturb some of the post-translational modifications observed in the region or may infer conformational changes that perturb the normal function of the protein.

## Data Availability

All data from this study that do not pertain to identifiable patient information are available and can be provided by contacting the corresponding author on reasonable request. The datasets used and/or analyzed during the current study are available from the corresponding author on reasonable request. The identified mutation has been submitted to the corresponding LOVD database (https://databases.lovd.nl/shared/individuals/00424916).

## List of Abbreviations

ACM: Arrhythmogenic cardiomyopathy
ACMG: American College of Medical Genetics and Genomics
ALVC: Arrhythmogenic left ventricular cardiomyopathy
CMR: Cardiac magnetic resonance
ECG: Electrocardiogram
GSR: Gly-Ser-Arg repeats
IF: Intermediate filaments
LBBB: Left bundle branch block
LGE: Late gadolinium enhancement
LV: Left ventricular
LVEF: Left ventricular ejection fraction
PRD: Plakin repeat domains
RBBB: Right bundle branch block
RV: Right ventricular
SCD: Sudden cardiac death
VPB: Ventricular premature beats
VUS: Variant of unknown significance
